# Antitumor compounds from *Streptomyces* sp. KML-2, isolated from Khewra salt mines, Pakistan

**DOI:** 10.1186/s40659-015-0046-3

**Published:** 2015-10-14

**Authors:** Usman Aftab, David L. Zechel, Imran Sajid

**Affiliations:** Department of Microbiology and Molecular Genetics, University of the Punjab, Quaid-e-Azam Campus, Lahore, 54590 Pakistan; Department of Chemistry, Queens University, Kingston, ON K7L 3N6 Canada

**Keywords:** *Streptomyces* sp. KML-2, Antitumor activity, Chromomycin SA, 1-(1*H*-indol-3-yl)-propane-1,2,3-triol

## Abstract

**Background:**

Actinomycetes are gram positive bacteria with high G + C content in their DNA and are capable of producing variety of secondary metabolites. Many of these metabolites possess different biological activities and have the potential to be developed as therapeutic agents. The aim of the present study was to screen actinomycetes inhabiting halophilic environment such as Khewra salt mines present in Pakistan for cytotoxic and antitumor compounds.

**Results:**

An actiomycetes strain designated as *Streptomyces* sp. KML-2 was isolated from a saline soil of Khewra salt mines, Pakistan. The strain *Streptomyces* sp. KML-2 showed 84 % cytotoxic activity against larvae of *Artemia salina*. In the screening phase, the strain exhibited significant antitumor activity with IC_50_ values of 12, 48 and 56 µg/ml against Hela, MDBK and Vero cell lines, respectively. After that extract from 20 l fermentation was used to purify secondary metabolites by several chromatographic techniques. Structure elucidation of isolated compounds revealed that it is highly stable producer of Chromomycin SA (1) and 1-(1*H*-indol-3-yl)-propane-1,2,3-triol (2). Both of the isolated compounds showed significant antitumor activity against Hela and MCF-7 cancer cell lines (IC_50_ values 8.9 and 7.8 µg/ml against Hela; 12.6 and 0.97 µg/ml against MCF-7, respectively). The 16S rRNA gene sequence (1437 bp) of the strain confirm its identity (99 %) with *Streptomyces griseus.*

**Conclusions:**

From this research work we were successful in isolating two potent antitumor compounds, Chromomycin SA and 1-(1*H*-indol-3-yl)-propane-1,2,3-triol from *Streptomyces* KML-2 strain, isolated from Khewra salt mine. As such this is the second report which confirms that *S. griseus* can produce Chromomycin SA without introducing any mutagenesis in its biosynthesizing gene cluster and isolated indole derivative is being reported first time from any member of actinomycetes group with having novel antitumor activity against Hela and MCF-7 cells.

Nucleotide sequences: Nucleotide sequence data reported are available in the GenBank database under the accession number: GenBank KJ009562.

**Electronic supplementary material:**

The online version of this article (doi:10.1186/s40659-015-0046-3) contains supplementary material, which is available to authorized users.

## Background

Nature is an attractive source of new therapeutic compounds, as it represents a pool of secondary metabolites with tremendous chemical diversity. Currently about 60 % of the approved therapeutic drugs are derived from natural sources. These sources include various animals, plants, marine-organisms, and microorganisms [1, 2]. Among the different microbial sources, actinomycetes specifically are famous as producers of great number of unique and chemically diverse bioactive compounds. Actinomycetes are filamentous, gram-positive, spore forming bacteria [3]. Members of the class actinomycetes are reported for the production of about 10,000 bioactive compounds among the total 23,000 bioactive secondary metabolites produced by microorganisms, representing 43 % of all bioactive microbial metabolites that have been discovered [4].

Actinomycetes are also known as a rich source of potent antitumor compounds. Regardless of the presence of several therapeutic methods for the cancer treatment, cancer is still a major public health threat [5]. Chemotherapy is one of the effective treatments for controlling cancer, but this approach needs a continuous supply of new antitumor compounds. Fortunately, one of the family members of actinomycetes known as *Streptomyces* helps us by providing promising antitumor compounds, which are unmatched and unrivaled in terms of treatment of cancer. The famous antitumor compounds produced by *Streptomyces* species and are being used in human chemotherapy includes actinomycin, mitomycin, anthracycline, bleomycin, aureolic acid families, pentostatin and resistomycin [6, 7]. Several studies confirm that *Streptomyces* are able to produce different antitumor compounds with diverse chemical backbones because they harbor different gene clusters encoding polyketide and nonribosomal peptide synthases [8]. Most of these compounds are secreted in the culture media and can be extracted using organic solvents [9]. There are many diverse mechanisms by which these compounds are able to control different tumor cells which includes apoptosis, mitochondrial permeabilization, blockage of signal transduction pathways by inhibiting key enzymes, cytomorphological changes due to disturbance in cellular differentiations, and tumor induced angiogenesis [10]. Sometime antitumor compounds isolated from *Streptomyces* strains act by intercalating with duplex DNA, which leads to detrimental effects on fast proliferating cells by inhibiting the DNA-dependent RNA polymerase activities [11].

In order to establish a unique pool of *Streptomyces* strains that can be examined for new anticancer drugs, we focused on gathering strains from unique habitats and conducting target directed biological screening [12, 13]. Among different habitats, normal terrestrial habitat is the most explored one, while there are very few studies conducted on extreme ecological niches, like forests, salt stress areas, hills, desert etc. [14]. Our aim from both of these strategies is to attain novelty in biological activities and chemical structures.

To this end, the present study reports the isolation of a *Streptomyces* strain, KML-2, from the Khewra salt mines in Pakistan. Culture extracts from KML-2 exhibited potent cytotoxic activity against *Artemia s.* larvae and antiproliferative activity against three cell lines. These activities inspired us to isolate the bioactive molecules and determine their structures and bioactivities. Phylogenetic analysis was performed by conducting ribotyping studies of the strain KML-2.

## Results and discussion

### Isolation and taxonomic characteristics of *Streptomyces* sp. KML-2

In order to meet the increasing demand for the treatment of human illness, new biologically active metabolites are needed. In this battle microorganisms are generally, and in particular actinomycetes, are prominent producers of chemically diverse compounds with multiple activities. However after several decades of extensive screening it has become difficult to simultaneously find novel compounds and novel microorganisms. Despite of this fact, there are some unique ecological niches in the world which are unexplored and could be a source of novelty, both for the microorganisms and their compounds. In our case we selected a unique and untapped source for the isolation of actinomycetes, the Khewra salt mine, which is the world’s second largest salt mine, located in the province Punjab, Pakistan [15]. Recently few studies report the isolation of novel actinomycetes strains from salt rich environments [16–18].

**Fig. 1:**
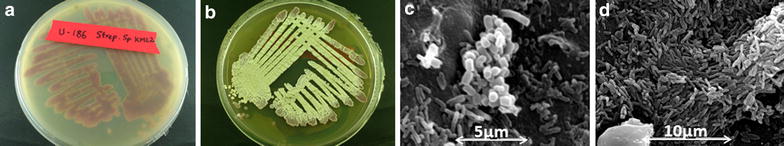
**a**, **b** Substrate and Aerial mycelia of *Streptomyces* sp. KML-2; **c**, **d** scanning electron microscope images of *Streptomyces* sp. KML-2

Several soil samples were collected from the mine and plated on the selective media after making suitable dilutions. After incubation actinomycetes strains were isolated and purified on GYM agar. The strain KML-2 exhibited typical *Streptomycetes* like characteristics (Table 1). Individual colonies grew well in an elevated manner with red vegetative mycelia and yellow aerial mycelia. As depicted in the scanning electron microscope image, the spore chains were straight, smooth, thick, and branched with very little coiling. The diameters of the spores are constant with having connection to substrate mycelia (Fig. 1a–d). All of the morphological and surface parameters observed under scanning electron microscope are in close similarity with family known as *Streptomycetaceae* and the genus *Streptomyces.*

**Table 1:** Taxonomic characteristics of the *Streptomyces* sp. KML-2

	Observed results
Morphological parameters	
Aerial mycelium	Detachable/pale yellow
Substrate mycelium	Abundant/red
Diffusible pigments	Nil
Fragmentation pattern of substrate mycelium	Fair
Spores	Abundant/smooth
Size of colony	3 mm
Consistency	Hard/embedded
Odour	Earthy
Elevation	Raised
Margins	Entire
Surface	Rough
Growth pattern	Well growth/partitioned
Shape/form	Circular
Optical feature	Opaque
Gram stain	Positive
Motility	Nil
Melanin production	−
Urease	++
Hemolysis	−
Utilization of sugars	
Glucose	+
Fructose	−
Arabinose	−
Galactose	+
Raffinose	−
Mannitol	+
Sucrose	−
Mannose	+
Utilization of organic acids	
Potassium gluconate	+
Trisodium citrate	+
Sodium malate	−
Sodium lactate	−
Sodium malonate	−
Oxalate	−

+, positive; −, negative; ++, strong positive

The comparison of biochemical, physiological and microscopic characteristics (Table 1) with those of known actinomycetes species mentioned in *Bergey’s Manual of Systematic Bacteriology* strongly suggested that the strain KML-2 belongs to the genus *Streptomyces* [19]. It can utilized d-galactose, d-glucose, mannose, glycerol and mannitol as a carbon source indicating its ability to grow on variety of sugars. The strain tested negative in biochemical tests like hemolysis and melanin production. Out of several organic acids tested for utilization, the strain KML-2 utilized only potassium gluconate and trisodium citrate (Table 1).

**Fig. 2:**
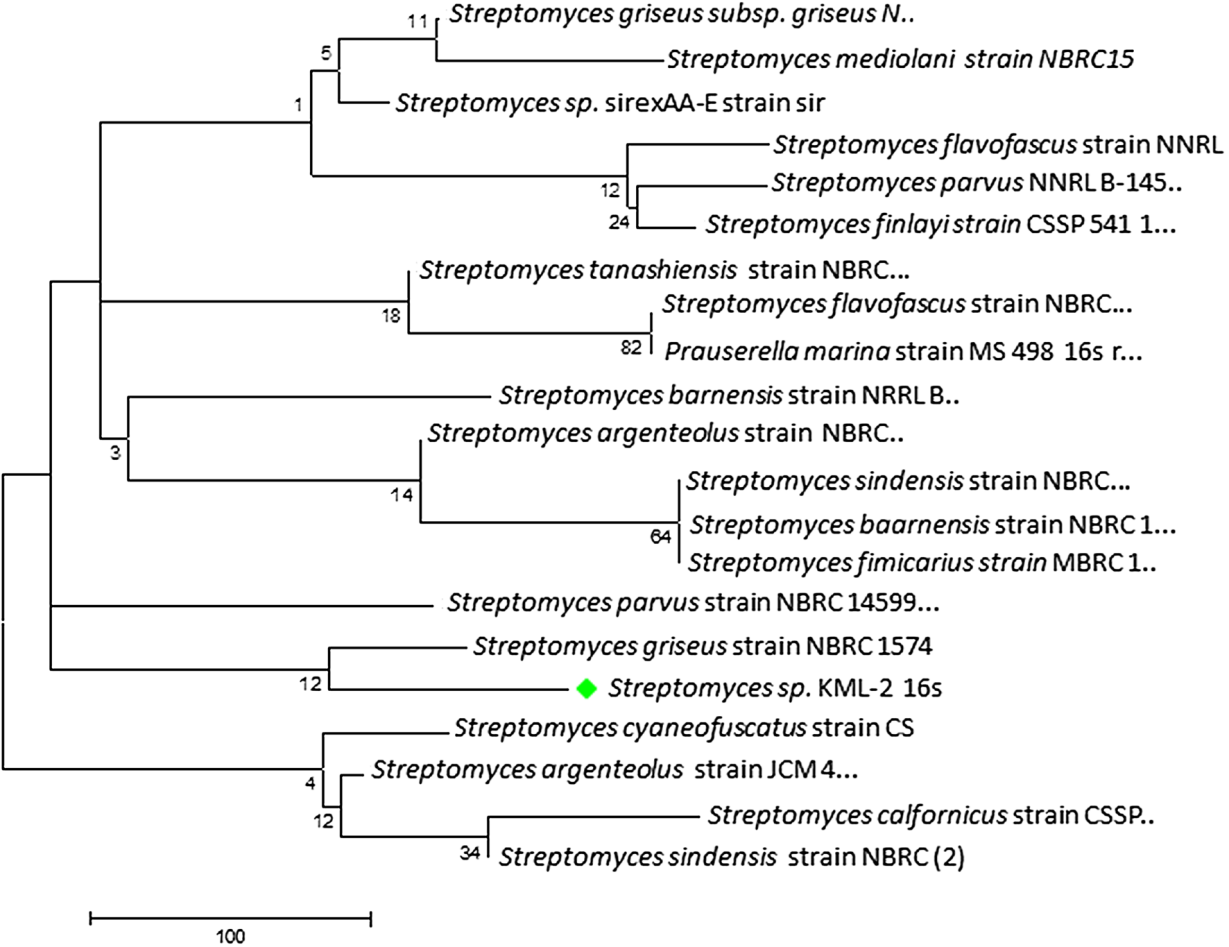
Phylogenetic tree of *Streptomyces* sp. KML-2

Several molecular techniques are being used in the research for investigating the evolutionary relationship among isolated microorganisms. By the help of these kinds of molecular techniques we can easily verify the hypothesis generated from other characterization methods like physiological and biochemical tests [20]. Among all the techniques ribotyping is very commonly used one, in which 16S rRNA gene sequence data of the isolated strain is compared with the sequence data present in the Genbank database. In our case we are able to sequence total nucleotide of 1437 bp (Accession No: KJ009562) of the 16S rRNA gene of *Streptomyces* sp. KML-2. The BLAST analysis was performed by aligning this sequence with the 16S rRNA gene sequences present in the Genbank database. Blast results exhibited highest similarity (99 % similarity) with the 16S rRNA gene of *Streptomyces griseus* (Fig. 2).

### Cytotoxicity and antitumor activity of *Streptomyces* sp. KML-2

A significant challenge in isolating bioactive compounds from microorganisms is having an appropriate and sensitive bioassay to guide the purification process [21]. In our case we screened extracts from *Streptomyces* sp. KML-2 using several bioassays in order to exploit its full chemical potential. We started with measuring the cytotoxic effects of the crude extract of this strain against the larvae of *Artemia s.* commonly known as brine shrimp [22]. The cytotoxic profile of the crude extract from *Streptomyces* sp. KML-2 showed very promising results. This strain produces compounds which have the ability to inhibit 84 % of the growth of *Atremia s.* larvae. The high cytotoxic response against *Artimia s.* suggests that the extract of this strain might contain some potent antitumor compounds. We then selected the MTT assay to test the antitumor activity of this strain against Hela cells (cervical cancer) and two normal cell lines including MDBK (cow kidney epithelial cells) and Vero cells (African green monkey kidney epithelial cells). We observed differential antitumor activity against tested cell lines in a dose-dependent manner. The survival rate of Vero and MDBK cells is higher compared to Hela cells. IC_50_ values for Hela, MDBK and Vero cells were 12.17, 47.88 and 56.12 µg/ml respectively. Such differential behavior with several normal and cancer cell lines including Hela were observed for the extracts and pure compounds isolated from different *Streptomyces* species in several studies [23–27]. These data indicate that the MTT assay utilized in our study is suitable for screening antitumor agents with high cytotoxic activities. No significant antibacterial activity was seen when the extracts was screened against the general panel of gram positive and gram negative bacteria.

**Fig. 3:**
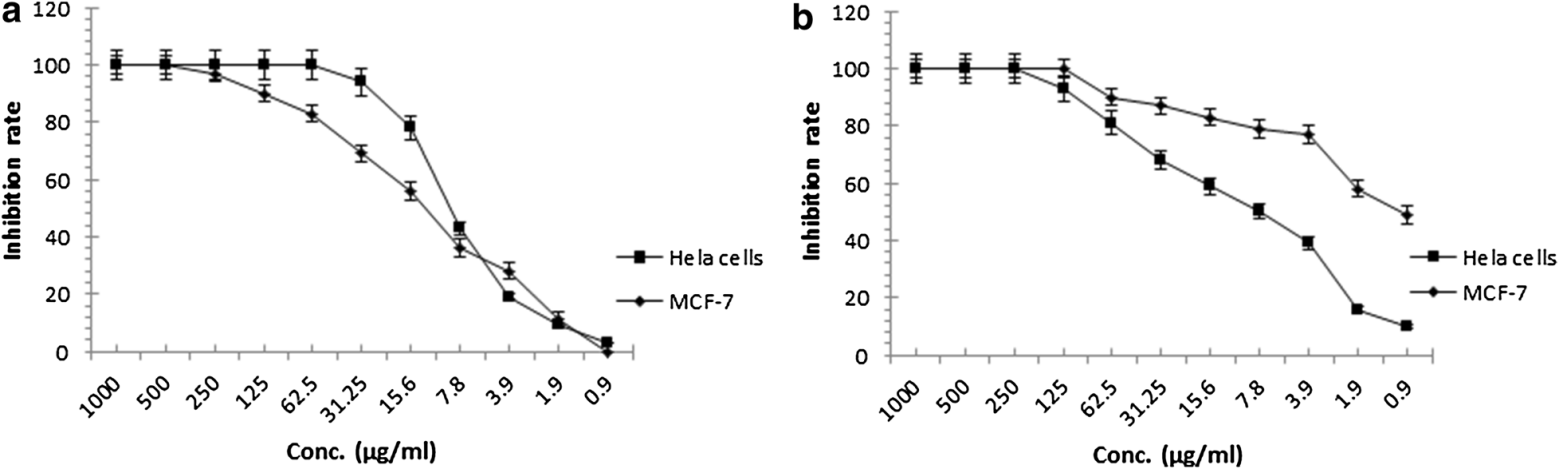
Activity of Compound 1 (**a**) and Compound 2 (**b**) against Hela and MCF-7 cell lines

Later on after isolation and purification, the antitumor activity of two pure culprit compounds was also evaluated by using MTT assay. Initially antitumor potential of the compound 1 and 2 was checked against Hela and MCF-7 (breast cancer) cell lines. It is observed that both compounds showed significant antitumor activity against both cell lines with IC_50_ value of 8.9 and 7.8 µg/ml against Hela cells respectively. Activity of compound 1 against MCF-7 is also significant with having IC_50_ value of 12.6 µg/ml, but compound 2, which was found novel in terms of source and activity after performing structure elucidation experiments, shows marvelous antitumor potential against MCF-7 cells with having IC_50_ value of only 0.97 µg/ml (Fig. 3).

### Purification and structure elucidation of the antitumor metabolites

**Fig. 4:**
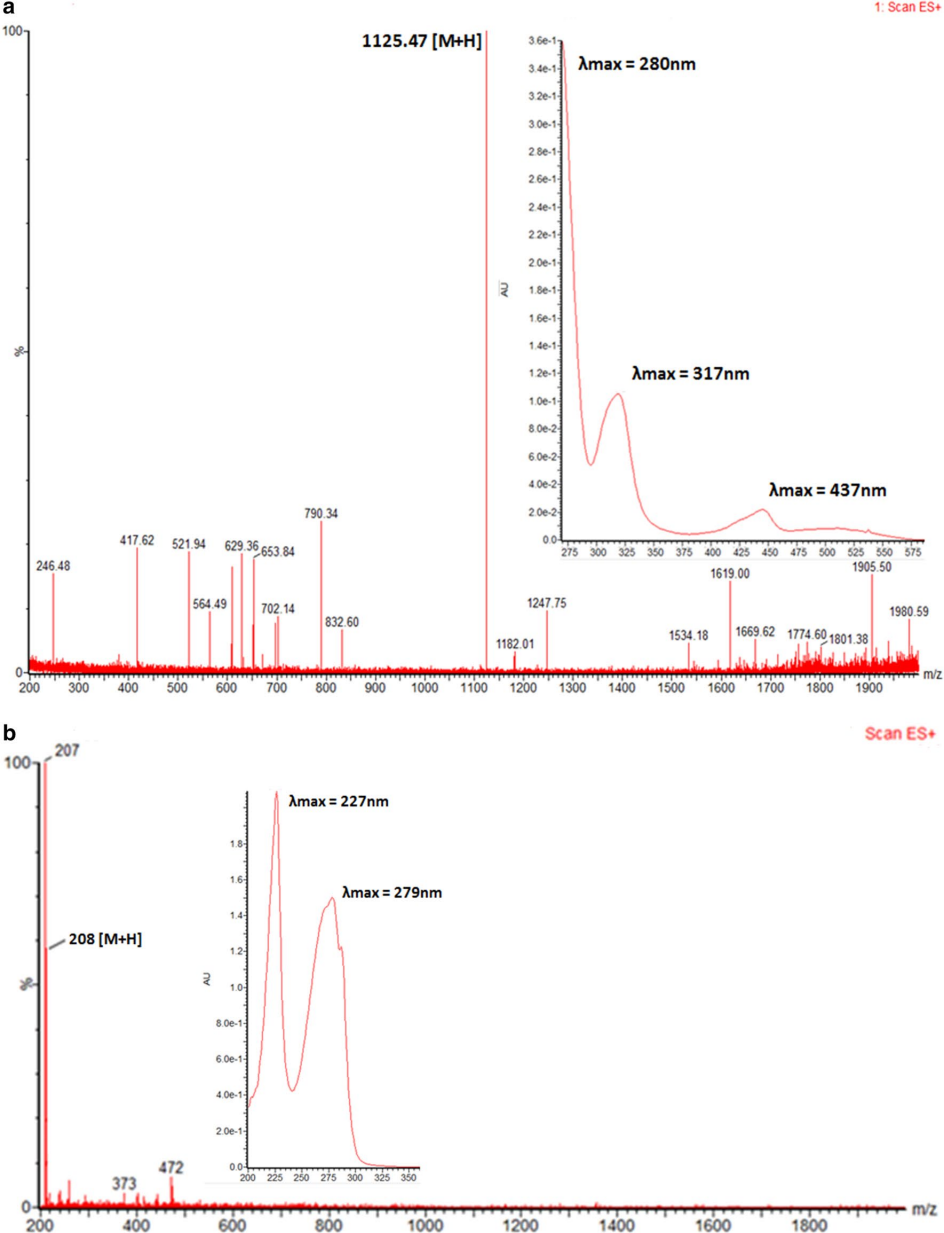
**a**, **b** Mass and UV spectra of compounds 1 and 2

**Fig. 5:**
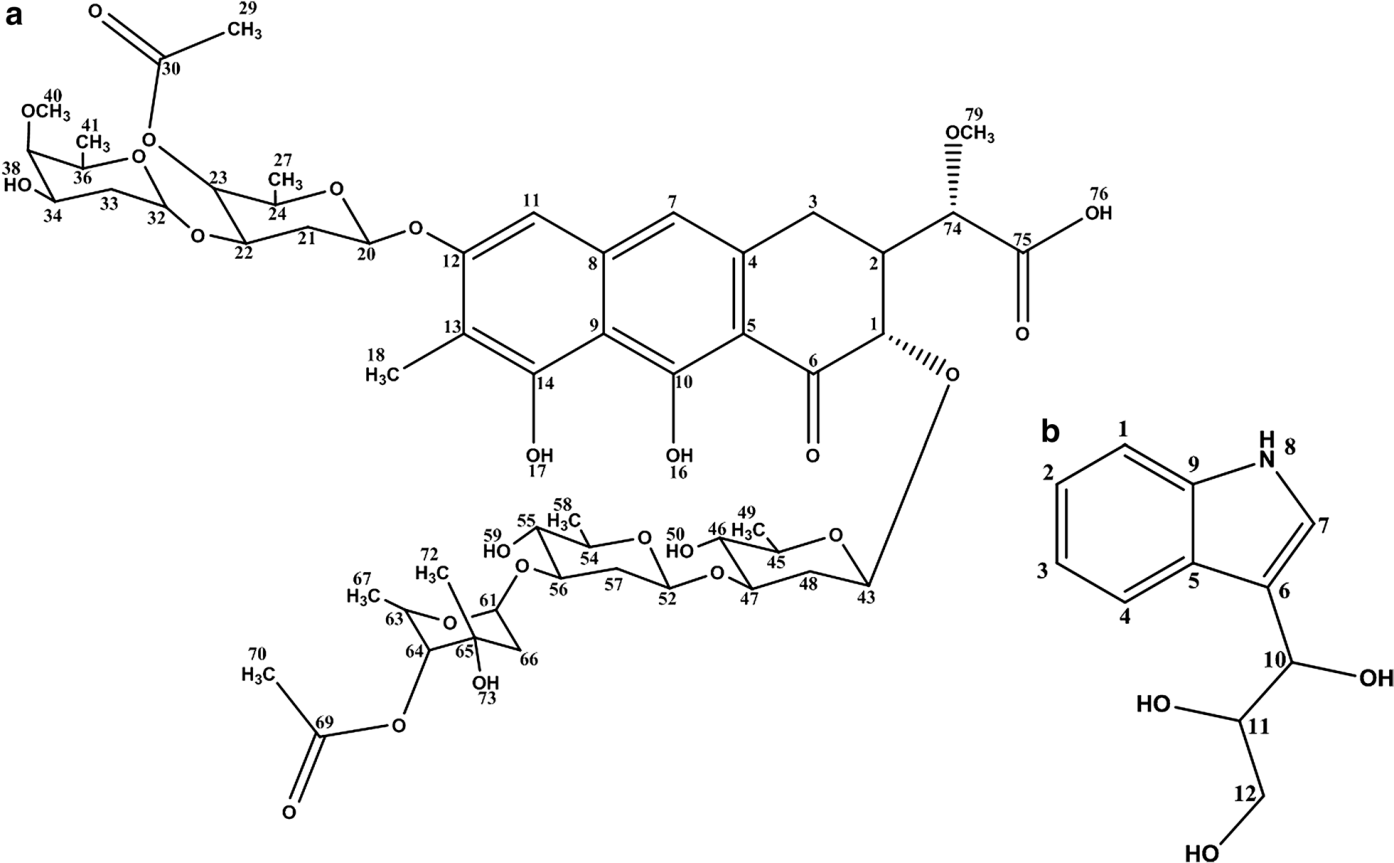
**a**, **b** Molecular Structure of Chromomycin SA (Compound 1) and 1-(1*H*-indol-3-yl) propane- 1, 2, 3-triol (Compound 2)

The 20 l shaking flasks culture of the isolate *Streptomyces* sp. KLM-2 was extracted using Diaion HP-20 beads and 1.5 g red color crude extract was obtained. Two different compounds were separated by preparative TLC. The compound 1 is yellow in color with λ_max_ at 437, 317 and 280 nm. After that this pure compound was analyzed on UPLC/MS system and *m*/*z* ratio of 1125.47 [M + H]^+^ was observed in positive ES mode. This value, along with the distinct absorbance spectrum is consistent with already reported compound named as Chromomycin SA having Chemical formula: C_54_H_76_O_25_, Exact mass: 1124.47, Molecular weight: 1125.17, Elemental analysis: C, 57.64; H, 6.81; O 35.55 (Figs. 4a, 5a). Results of ^1^H-NMR experiment for this isolated compound confirmed its identity as Chromomycin SA (Additional file 1: Table S1). Chromomycin SA is a yellow color compound produced by *Streptomycesgriseus*, already reported for a potent activity against Hela cells [23]. Our phylogenetic, morphological and physiological analysis also confirm that our strain have 99 % similarity with *S. griseus*. Chromomycin SA belongs to the class of polyketide compounds known as aureolic acids. Aureolic acid family contains potent antitumor compounds which inhibit the replication and transcription process during macromolecules biosynthesis. They also interact with the minor groove of DNA in GC-rich region. This binding is non-intercalative in nature, via cross linking of DNA in the presence of Mg^2+^ [28].

Previously Menéndez et al. [23] reported isolation of Chromomycin SA from a *S. griseus* strain that has been genetically altered specifically through the inactivation of ketoreductase gene (*cmmW1*). Later on Hu et al. [29] reports the isolation of Chromomycin SA without introducing any mutation in ketoreductase gene. They proposed that Chromomycin SA is byproduct in nature, formed during the synthesis of different analogs of Chromomycin SA. They have isolated the *S. griseus* strain from saline environment which is also similar in our case. So we can hypothesized that might be there is some relation between inactivation of ketoreductase gene and stress acclimatization. Latest studies have also introduced the new term known as ecovars, in which strains of *S. griseus* might be undergoing ecology-specific evolution, which results in the genetic variation with the specific ecology [30].

Compound 2 with *m*/*z* 208 [M + H]^+^ was purified by column chromatography over silica gel with the Methanol: Dichloromethane solvent gradient system and Sephadex LH-20 column. After purification 6 mg of pure compound was obtained. Structure of this pure compound was determined through Bruker Avance 600 MHz NMR spectrometer. After performing several two dimensional NMR experiments compound 2 was identified as 1-(1*H*-indol-3-yl) propane- 1, 2, 3-triol (Fig. 4b), one of the derivative of indolic compounds (Additional file 1: Table S2) with Chemical formula: C_11_H_13_NO_3_, Exact mass: 207.09, Molecular weight: 207.23, Elemental analysis: C, 63.76; H, 6.32; N, 6.76; O, 23.16. To the best of our knowledge this type of indolic derivative has not been reported earlier from *Streptomyces* source, however one study depicted its isolation from clavicipitaceous fungi *Balansia epichloe* (*B. epichloë*) with high toxicity against fertile chicken eggs [31]. Earlier only fungus is reported among natural sources for the production of this type indolic derivative but now some studies also reports its chemical synthesis [32]. Several actinomycetes strains were reported for the presence of tryptophan metabolism gene clusters, which are responsible for the production of different indolic derivatives [33]. As shown in Fig. 4b 1-(1*H*-indol-3-yl)-propane-1,2,3-triol has λ_max_ at 227 and 279 nm, which is very common among different derivatives of indoles which are reported for having promising antitumor activity against several different tumor cell lines [34].

## Conclusion

From this research work we were successful in isolating two potent antitumor compounds from *Streptomyces* KML-2 strain. From the results we can inferred that the strain *Streptomyces* KML-2 is potent source of antitumor agents. This study also reveals that Khewra salt mine from where the strain KML-2 was isolated is a potent ecological niche with having inimitable strain diversity which are yet to be discovered. In short these unique habitats should be continuously explored for extracting lead antitumor compound. Furthermore we will try to check the activity of isolated compounds against different cancers including colorectal and liver cancers, which are present in very high intensity worldwide and specifically in Pakistan.

## Methods

### Sample collection and enrichment

Soil samples were collected from Khewra salt mines, Punjab, Pakistan, in sterile sampling bag, from the depth of 5–10 cm, quantity of each of the collected sample was between 2 and 5 g. The samples were then treated using different physical and chemical methods for the enrichment of actinomycetes. In physical treatment the samples were kept at high temperature (55 °C) for 2–3 weeks and in chemical treatment soil samples were mixed with CaCO_3_ at the ratio of 10:1 and were incubated under moisture rich conditions for 7 days at room temperature [35].

### Selective isolation of actinomycetes

Actinomycetes strains were isolated using selective isolation media Glycerol-Casein-KNO_3_ agar (glycerol 10 g, KNO_3_ 2 g, casein 0.3 g, NaCl 2 g, K_2_HPO_4_ 2 g, MgSO_4_-7H_2_O 0.05 g, CaCO_3_ 0.02 g, FeSO_4_-7H_2_O 0.01 g, agar 18 g, all in 1 l) containing cycloheximide (50 μg/ml) as antifungal agent [36]. About 1 g of soil was dissolved in autoclaved distilled water and serial dilutions (10^−2^ to 10^−4^) were plated on Glycerol-Casein-KNO_3_ agar. The plates were incubated for 7–21 days at 28 °C. The actinomycetes colonies were selected and purified by repeated sub-culturing on GYM agar (10 g malt extract, 5 g yeast extract, 5 g glucose, 15 g agar, all in 1 l of tap water) [37]. Later the selected isolates were preserved in liquid nitrogen for future use.

### Morphological, biochemical and physiological characterization of the isolated strain

The morphologic, biochemical and physiological characteristics of the isolate KML-2 were studied by the methods employed in International *Streptomyces* Project (ISP), including colony size, consistency, shape, elevation, margins, color of aerial/substrate mycelium, pigments diffusing into the medium, formation of melanin, utilization of different sugars as sole source of carbon, utilization of organic acids, hemolysis, utilization of oxalates, optimum pH, temperature, aeration, media composition [37, 38].

### Scanning electron microscopy (SEM)

In order to see the deep morphological pattern (substrate & aerial mycelia) of strain KLM-2, scanning electron microscopy was performed [39]. The strain was grown on GYM agar and a piece of agar containing sufficient growth of the strain was cut with sterile scalpel. The strain was fixed with glutaraldehyde (2 %) and formaldehyde (5 %), and then incubated at 4 °C for overnight in 0.1 M sodium cacodylate buffer (0.1 M cacodylate, 0.01 M CaCl_2_, 0.01 M MgCl_2_, 0.09 M sucrose; pH 6.9). After overnight fixation, the strain was washed with fresh cacodylate buffer. The strain was then dehydrated with a series of acetone solutions (10, 30, 50, 70, 90, and 100 % acetone). After acetone dehydration, the agar piece containing growth was subjected to critical-point drying with liquid CO_2_. Before loading the agar piece in the scanning electron microscope (SEM) it was covered with a 10 nm thick gold film by sputter coating (Hummer-V Sputter Coater). After coating, deep morphological patterns of the substrate mycelia of strain KML-2 were analyzed by using scanning electron microscope (HITACHI S-2300 SEM at Electron microscopy facility, Queens University, Canada).

## 16S rRNA gene sequencing

Total genomic DNA was isolated after growing the stain in GYM broth. Genomic DNA was extracted by the phenol/chloroform method as described by Hopwood et al. [40]. The 1.5 kb 16S rDNA fragment was amplified by PCR using the universal primers 27F, 5′-AGAGTTTGATCCTGGCTCAG-3′ and 1522R, 5′-AAGGAGGTGATCCARCCGCA-3′. Each PCR reaction vial contained approximately 300 ng genomic DNA, 2 µl of each primer having working concentration of 10 pmol, and 25 µl of 2X PCR master Mix (Merck-GeNei™). The reaction was cycled as follows: 94 °C for 5 min; 30 cycles of 94 °C for 20 s; 50 °C for 20 s; 72 °C for 2 min; followed by 72 °C for 5 min. After amplification, the reaction product was resolved by agarose gel electrophoresis and gel purified using a MiniElute™ PCR purification kit (Qiagen, USA). The gene product was sequenced using dye terminator chemistry on an automated sequencer (ABI-PRISM^®^ BigDye^®^ Terminator version 3.1 Cycle Sequencing Kit, Applied Biosystems, USA), and the sequence data was compared to existing sequences by BLAST analysis (http://www.nih.nlm.gov/blast.cgi). After analyzing BLAST results, phylogenetic and molecular evolutionary analysis were conducted using software *MEGA* (version 4) [41]. The sequence was then deposited to the NCBI GenBank.

### Small scale cultivation of strain KML-2

The *Streptomyces* strain KML-2 was grown in 250 ml GYM broth in 1 l Erlenmeyer flasks (the pH was adjusted to 7.8 before sterilization). The flasks were then incubated at 28 °C at 100 rpm for 5–7 days. After incubation the cells were disrupted by sonication. The resulting broth was mixed with ethyl acetate 1:1. The mixture was sonicated again and the upper organic layer of ethyl acetate was collected in a separatory funnel. The organic layer was evaporated on rotary vacuum evaporator (Heidolph^®^ 4000 efficient) and a small amount of crude extract was obtained. The crude extract was analyzed for cytotoxic and antitumor activities against different cell lines.

### Determination of cytotoxic and antibacterial activity of *Streptomyces* sp. KML-2

A microwell cytotoxicity assay as described by Solis et al. [42] was used to check the cytotoxicity of the crude extract against brine shrimp larvae (*Artemia s.*). In order to calculate the mortality of the larvae, dried eggs of *Artemia s.* (0.5 g) were added to a 500 ml separating funnel filled with 400 ml of artificial seawater and were incubated at room temperature for 48 h under extensive aeration. After incubation the whole suspension was kept undisturbed for 1 h in order to allow the eggs to settle down. Active larvae were collected by exposing one side of the extraction funnel with light source. About 30–40 active larvae were transferred to the wells of microtiter plate (wells diameter 1.8 cm, depth 2 cm) already filled with 0.2 ml of salt water. Dead larvae were counted (value N) before adding 20 µg of the crude extract in 5 µl of DMSO. The plate was kept at room temperature in the dark. After 24 h, the number of the dead larvae (value A) in each well was counted under the microscope. In order to calculate total number of larvae (value G) all of surviving larvae were killed by the addition of 0.5 ml methanol. The test was performed in triplicate; each test row was accompanied by a blind sample containing pure DMSO as negative control. Actinomycin-D (10 µg/ml) was used as a positive control with 100 % mortality rate. Mortality rate was calculated by using the following formula:

$${\text{M }} = \, \left[ {\left( {{\text{A }}{-}{\text{ B }}{-}{\text{ N}}} \right)/\left( {{\text{G }}{-}{\text{ N}}} \right)} \right] \, \times { 1}00$$

where, M = Percent of the dead larvae after 24 h, A = Number of the dead larvae after 24 h, B = Average number of the dead larvae in the blind samples after 24 h, N = Number of the dead larvae before starting of the test, G = Total number of larvae.

Antimicrobial activity of the extract obtained from the culture broth of *Streptomyces* sp. KML-2 was determined by disc diffusion method as described by Sajid et al. [43] against a set of test organisms including *Bacillus subtilus*, *E. coli* (ATCC 25922), *Staphylococcus aureus* (ATCC 25923), *Methicilin resistant Staphylococcus aureus* (*MRSA*), *Acinetobacter*, *Pseudomonas aeruginosa and klebsiella pneumonia* (ATCC 706003).

### In-vitro screening for antitumor activity

The antitumor assay was performed on Hela, MDBK, Vero and MCF-7 cell lines (ATCC) using MTT assay [44]. The cells were grown in 96 well plate in Dulbecco’s modified Eagle’s medium (Invitrogen, NY, USA) supplemented with 10 % fetal bovine serum and 1 % antibiotics (streptomycin and penicillin-G) by incubating at 37 °C for 24 h in 5 % CO_2_ (Sanyo CO_2_ incubator MCO-15AC) under humid conditions. After the formation of confluent monolayer of the actively dividing cells, trypsinization was done and cell suspension (10^5^ cells/ml) was then seeded in the wells containing culture media and different concentrations of the extract obtained from the shaking culture of KML-2 strain. Plate was then incubated at 37 °C for 48 h in 5 % CO_2_ environment. After incubation cellular viability for each concentration of the extract was measured as described by Mosmann, where in 20 µl of MTT (5 mg/ml) was added to each well and the plate was again incubated at 37 °C for 3 h in 5 % CO_2_ environment. After incubation the media was carefully removed and 100 µl of DMSO was added in order to solubilize the formazan crystals produced by metabolically active cells. The optical density (O.D) of the wells were then measured with a microplate reader (Epoch BIOTEK^®^) at 570 nm with 655 nm as reference wavelength. Cellular images were taken through inverted microscope (LABOMED iVu3000-TCM400). Assay controls were maintained throughout the experiment and the assay was performed in triplicates. Calculations of IC_50_ were done through dose dependent curve. The growth inhibition rate for each dilution was calculated by the following formula:

$${\text{Inhibition rate = }}\frac{{{\text{O}}.{\text{D }}\left( {\text{control well}} \right) \, - {\text{ O}}.{\text{D }}\left( {\text{treated well}} \right)}}{{{\text{O}}.{\text{D }}\left( {\text{control well}} \right)}}\; \times \; 100$$

### Fermentation and extraction

A starter culture was prepared by inoculating 25 ml GYM broth with the strain KML-2 and incubating at 28 °C for 4 days at 95 rpm. Fermentation was done in ten 5 l EM baffled flasks containing springs and glass beads. Each flask contained 2 l GYM broth for a total working volume of 20 l. Antifoaming agent 204 (Sigma Aldrich), 100 µl/l, and 2 % (v/v) inoculum was added to each flask. The cultures were incubated at 28 °C for 7 days at 95 rpm. After 7 days the culture broth was harvested by centrifugation at 11,000 rpm for 5 min at ambient temperature. After centrifugation, the liquid phase was separated and Diaion^®^ HP-20 beads (Sapelca analytical USA), pre-washed three times with acetone and air dried overnight, were added at a ratio of 10 g/l. This mixture was incubated for 3 h on a rotary shaker at 100 rpm, and then separated by vacuum filtration. The beads were washed with water 2–3 times followed by three times washing with acetone. The acetone was removed under vacuum to dryness. The solid phase or cellular mass was also extracted three times with ethyl acetate and the solvent was evaporated under vacuum to dryness.

### Purification and structure elucidation by UPLC–MS and NMR analysis

The combined crude extract (extract from liquid as well as solid phase) was fractionated on silica gel column (25 × 5 cm, Silica gel 60, Merck). The fractions were purified by repeated silica gel column chromatography and preparative TLC (PTLC) with a mobile phase of CH_2_Cl_2_/MeOH gradient. Final finishing of the purified compounds was done by passing the fractions through Sephadex LH-20 column. The structures of the purified compounds were analyzed through UPLC-mass spectrometry and NMR spectroscopy. The data obtained was compared with reference data present in databases including: Dictionary of Natural Products (DNP), Pub Chem, Scifinder and Chemical abstracts. Compound 1 and 2 were isolated and characterized by UPLC-MS and NMR spectroscopy. For UPLC-MS analysis samples were prepared carefully by dissolving the powder compound in 500 µl methanol. Furthermore this methanolic compound mixture was five times diluted in 50 % acetonitrile. The diluted samples were then filtered through 2 µm pore size disposable syringe filters and further centrifuged at 14,000 rpm in order to remove any particle. Samples were then transferred to special LC–MS glass vials which were further placed in the sample tray of UPLC-MS machine. The analytical conditions for the UPLC-MS analysis were as follows: Waters ACQUITY UPLC H-class BEH-C18, (2.1 × 100 mm; particle size, 1.7 μm) column; Column temperature: Ambient; Gradient Elution: A is Acetonitrile, B is Acetonitrile + 0.5 % acetic acid, C is MilliQ water + 0.5 % acetic acid, D is MilliQ water; Gradient Profile: 0–1.5 min 5 % B and 95 % C, 1.5–7 min is 95 % B and 5 % C, 7–10 min 95 % B and 5 % C, Before and after run column was re-equilibrate by eluting with 100 % A and B; Flow rate: 0.5 ml/min; *m*/*z* monitoring range 200–2000 *m*/*z* ratio; Wavelength monitoring range 220–800 nm; Total run time: 10 min; Data: Continuum; Seal wash period: 5 min; Waters SQ detector mass spectrometer; Ionization mode: ESI positive; Scan Duration: 0.5 s; Capillary voltage, 3.3 kV; Cone voltage ramp: on; Source temperature: 150 °C; Desolvation temperature: 400 °C; Desolvation gas flow: 800 l/h; Cone voltage; 0 V; Desolvation gas: liquid nitrogen; Mass lynx software V 4.1 (waters).

Structure of the compounds were elucidate on Bruker Avance 600 MHz NMR machine by using simple 1D and 2D NMR spectroscopic techniques for ^1^H- and ^13^C. Several techniques including COSY, HMBC, HSQC and NOESY were also performed for the correct structure elucidation in the 2D NMR spectroscopic analysis.

## Additional file

10.1186/s40659-015-0046-3 Table S1: NMR correlation table of compound 1. Table S2: NMR correlation table of compound 2.
