# Supplementary material for: Antitumor compounds from Streptomyces sp. KML-2, isolated from Khewra salt mines, Pakistan
Source: Biol Res. 2015 Oct 14;48:58. doi: 10.1186/s40659-015-0046-3 (PMC4607010; doi:10.1186/s40659-015-0046-3)
Supplement: Supplementary file 1 — 10.1186/s40659-015-0046-3 Table S1: NMR correlation table of compound 1. Table S2: NMR correlation table of compound 2. [file 40659_2015_46_MOESM1_ESM.docx]

**SUPPLEMENTARY MATERIAL**

**Antitumor compounds from *Streptomyces* sp. KML-2, isolated from Khewra salt mines, Pakistan**

Usman Aftab^a,b,*^, David L. Zechel^b^ and Imran Sajid^a^

*^a^Department of Microbiology and Molecular Genetics, University of the Punjab, Quaid-e-Azam Campus, Lahore 54590, Pakistan; ^b^Department of Chemistry, Queens University, Ontario, Kingston, K7L 3N6, Canada.*

**Table of Contents**

**Table S1:** NMR CORRELATION TABLE OF COMPOUND **1**

**Table S2:** NMR CORRELATION TABLE OF COMPOUND **2**

**Table S1.** NMR Correlation Table of compound 1 (Chromomycin SA)

Molecule in CD_3_OD

Ref. CD_3_OD: ^1^H-NMR 3.31 ppm

| Position | δ H (ppm), mult | J (Hz) | Position | δ H (ppm) ,mult | J (Hz) |
| --- | --- | --- | --- | --- | --- |
| 1  2 | 4.41 (d)  3.45(m)overlap | 11.6  -- | 46 | 3.73 (dd) | 8.5,8.5 |
| 3 | 2.59 (dd)  2.81 (d) | 16.3,3.4  15.4 | 47 | 3.13 (m) | -- |
| 7 | 7.82 (s) | -- | 48 | 1.99 (dd)  1.78 (dd) | 12.0,5.0  12.0, 12.0 |
| 11 | 6.96 (s) | -- | 49 | 1.26 (d) | 6.2 |
| 18 | 2.33 (s) | -- | 52 | 5.01 (dd) | 9.2,1.9 |
| 20 | 5.93 (m) | 9.8,2.2 | 54 | 3.96 (m) | -- |
| 21 | 2.19 (m)  1.99 (dd) | --  10.5,4.0 | 55 | 3.73 (dd) | 8.5,8.5 |
| 22 | 3.64 (m) | -- | 56 | 3.13 (m) | -- |
| 23 | 4.51 (d) | 2.6 | 57 | 1.98 (dd)  1.71 (d) | 12.0,5.0  12.0 |
| 24 | 4.43 (q) | 6.6 | 58 | 1.28 (d) | 6.1 |
| 27 | 1.23 (d) | 6.6 | 61 | 5.05 (t) | 2.4 |
| 29 | 2.25 (s) | -- | 63 | 4.44 (m) | -- |
| 32 | 5.09 (br s) | -- | 64 | 4.22 (d) | 9.2 |
| 33 | 2.00 (dt)  1.77 (m) | 12.2,3.8  -- | 66 | 1.98 (m)  1.76 (dd) | --  13.6,3.8 |
| 34 | 3.77 (m) | -- | 67 | 1.26 (d) | 6.2 |
| 35 | 3.11 (d) | 2.6 | 70 | 2.25 (s) | -- |
| 36 | 4.19 (q) | 6.5 | 72 | 1.35 (s) | -- |
| 40 | 3.39 (s) | -- | 74 | 3.79 (br s) | -- |
| 41 | 1.29 (d) | 6.5 | 79 | 3.39 (s) | -- |
| 43 | 5.06 (dd) | 9.9,1.7 |  |  |  |
| 45 | 3.93 (m) | -- |  |  |  |

# Table S2. NMR Correlation Table of compound 2 (1-(1H-indol-3-yl) propane- 1, 2, 3-triol)

# Molecule in CD_3_OD

Ref. CD_3_OD:^1^H-NMR 3.31 ppm, 49.0 for ^13^C

| Position | δ^1^H (ppm), mult | J (Hz) | ^13^C | HMBC | NOESY |
| --- | --- | --- | --- | --- | --- |
|  |  |  |  |  |  |
| 1 | 7.35 (dt) | 8.1, 0.8 | 112.2 | 3, 5 |  |
| 2 | 7.09 (ddd) | 8.1, 7.0, 1.1 | 122.2 | -- |  |
| 3 | 7.01 (ddd) | 7.9, 7.0, 0.9 | 119.6 | 1, 5 |  |
| 4 | 7.70 (dt) | 8.1, 0.9 | 120.1 | 2, 5, 6, 9 |  |
| 5 | -- | -- | 127.5 | -- |  |
| 6 | -- | -- | 116.1 | -- |  |
| 7 | 7.28 (s) | -- | 123.9 | 5, 6, 9, 10 |  |
| 8 NH | -- | -- | -- | -- |  |
| 9 | -- | -- | 137.9 | -- |  |
| 10 CH-OH | 5.01 (d) | 6.0 | 70.5 | 5, 6, 7, 11, 12, |  |
| 11 CH-OH | 4.05 (td) | 6.3, 3.8 | 75.9 | 6, 10, 12 |  |
| 12a CH2-OH  12b | 3.76 (dd)  3.66 (dd) | 11.3, 3.8  11.2, 6.7 | 64.5  -- | 10  10, 11 | - *7***, 10, 11, 12b** - *7*, **10, 11, 12a** |

- Note: Bold mean strong and italic (not bold) mean weak noe
